# The Use of Binaural Based Spatial Audio in the Reduction of Auditory Hypersensitivity in Autistic Young People

**DOI:** 10.3390/ijerph191912474

**Published:** 2022-09-30

**Authors:** Daniel Johnston, Hauke Egermann, Gavin Kearney

**Affiliations:** 1 AudioLab, Communication Technologies Research Group, School of Physics, Engineering and Technology, University of York, York YO10 5DD, UK; 2 Institute of Music and Musicology, TU Dortmund University, Emil-Figge-Straße 50, 44227 Dortmund, Germany

**Keywords:** autism spectrum disorders, virtual reality, auditory processing, auditory hypersensitivity, tools for therapy, multisensory, spatial audio, serious games

## Abstract

Individuals diagnosed with autism spectrum disorder (ASD) are characterised as experiencing impairments in social-emotional interaction and communication, alongside frequently displaying repetitive behaviours and interests. Further to this, they are often described as experiencing difficulties in processing sensory information, with particular prevalence within the auditory modality. Provoked by common environmental sounds, auditory hypersensitivity can result in self-regulatory fear responses. Rather than a physiological pain reaction, literature suggests that these hypersensitivities are resulting through irrational fear of the sounds. This investigation evaluates the use of binaural based spatial audio as a rendering technique for delivering realistic simulations of averse stimuli within a virtual reality (VR) exposure based computer game intervention for auditory hypersensitivity in autism. Over multiple experimental sessions, 20 autistic participants experiencing auditory hypersensitivity were exposed to either spatial audio or stereo renders of target stimuli during the intervention. Measurements of self-reported emotions displayed significant reductions in associated negative emotional reactions to target stimuli for all participants. However, significant improvements were experienced by those listening to spatial audio simulations. Moreover, tracked voluntary interactions with exposure based game-mechanics increased as the study progressed. Providing further evidence of increased tolerance towards averse auditory stimuli.

## 1. Introduction

Autism spectrum disorder (ASD) is a complex, pervasive neurological disorder that occurs in approximately 1% of the worlds population. With those diagnosed with autism characterised through impaired social communication development and repetitive behaviours and interests [1]. Further to this, many autistic individuals are described as having atypical responses to sensory information, with sound sensitivity being a specifically poignant and prevalent issue. Data compiled by the Autism Research institute from over 17,000 families of autistic children observed that ≈40% of parental questionnaires reported auditory hypersensitivities [2]. Often provoked by common everyday sounds, individuals can display sound-avoidance behaviours which may be observed as autonomic fear responses such as covering the ears, vocalisation and fleeing the area. These reactions to perceptually averse auditory stimuli will have an impact upon the social and communication development of the individual, as a result of activity limitations, self-isolation and participation restrictions brought on by sound avoidance behaviours [3].

The causes of auditory hypersensitivity have been the subject of much research which has aimed to identify physiological evidence that would demonstrate discrepancies in auditory processing between autistic people and their typically developed (TD) peers. However, investigations focusing on auditory thresholds and tolerance to loudness found no significant differences between autistic participants and TD controls [4,5,6]. Furthermore, rather than a generic response to sounds of a particular intensity or frequency, empirical evidence also suggests that negative behaviours are activated by specific sounds [3,7,8,9]. With this in mind, researchers have suggested that rather than an auditory based problem, adverse reactions may represent psycho-emotional responses caused by impairments in the limbic system [6,9,10]. If left untreated, individuals will continue to display negative emotional reactions and sound avoidance behaviours which can have a significant effect on the quality of life of themselves and family [7]. Therefore, it is suggested that early interventions are crucial in order to reduce any long lasting negative impacts [3].

Intervention strategies utilising ear protection and noise cancelling headphones can provide a temporary solution to auditory hypersensitivity by attenuating auditory stimulation [11,12]. Cognitive behavioural therapy (CBT) interventions such as systematic desensitisation and graded exposure have however demonstrated long term success in reducing fear based responses to auditory stimuli. An early example of which is a case study by Jackson & King [13] in which a desensitisation process was used to reduce phobic responses to the sound of a toilet flushing in a 4-year-old autistic boy. An in vivo exposure desensitisation technique was used in which the child would be tickled under the arm whilst using the toilet and flushing. If no negative behaviours were displayed, the child would receive verbal praise and an edible. Following a 15 day programme, the phobic reactions to the toilet flushing were eliminated and these results were maintained at three and six month follow ups.

Another example of desensitisation successfully used to reduce auditory hypersensitivity in autistic children was conducted by Koegal et al. [7]. The investigation involved a group of three children who exhibited extreme aversion to sounds including toilet flushing, house hold appliances and animal noises. All participants displayed extreme behaviours such as sound avoidance, covering the ears and screaming. The systematic desensitisation process involved a mixed hierarchical exposure towards real-world stimuli and sounds played through speakers during play activities. At each stage of the intervention the sound source would move closer to the child. When compared to baseline measurements all participants showed a considerable decrease in anxiety levels and adverse reactions to auditory stimuli following the intervention period. This resulted in the children being able to tolerate the sounds and display some positive behaviours, for example laughing and smiling. Another important outcome from this study is that the extinction of negative behaviour was maintained at a 34 week follow-up measurement.

Despite extensive literature providing empirical evidence in support of CBT as a therapeutic technique to reduce phobic responses to stimuli [14], there are a number of augments that indicate CBT is not appropriate for individuals with ASD. Firstly, CBT sessions require face-to-face communication in order to teach the individual to become self-aware of a pathological response and to construct new positive associations with the stimulus [15]. For an individual with social and communication impairments, this may result in a diminished motivation to engage with an intervention [16]. Further to this, many CBT approaches that utilise imaginal exposure are not contextualised and often do not fully represent the medium in which the patient will experience the anxiety. This can be problematic for autistic individuals, as they require frequent and contextualised exposure to maximise the opportunity for generalisation [17]. Finally, another noteworthy consideration of current CBT is a shortfall in accessibility to treatment. A systematic review conducted by Ince et al. [18] indicated that the rates of implementation for CBT within the United Kingdom are below the recommended levels. This has been attributed to a lack of resources, limited dedicated therapy time and a shortage in specialist training [19].

Increasingly, computer based interventions such as serious games (SG), which aim to deliver therapy via a computer game modality, are being utilised to augment traditional psychological treatments for ASD [20,21]. These applications are capable of integrating CBT mechanisms into the core game-mechanics and loops, creating a safe, controllable and engaging environment in which players can repeatably practise newly acquired skills. In addition, serious games offer a cost-effective and accessible approach to therapy for those diagnosed with ASD. One such game developed for auditory hypersensitivity for ASD is *Sinbad and the Magic Cure,* in which players must voluntarily expose themselves to adverse sounds in order to progress the game-play [22].

One key technology currently being utilised to deliver computer based interventions is Virtual Reality (VR), a platform that has the capacity for rendering realistic three dimensional audio and visual environments [23]. This has been utilised in the treatment of specific phobias in autistic young people by Maskey et al. [24]. Throughout the study with the virtual reality environment (VRE), participants were exposed to hierarchical levels of a simulated feared stimulus, including bees, dogs and open spaces. Following the multi-session intervention, one-third of participants displayed improvements in their real-world phobias, taking part in activities and situations that were not previously possible. A similar approach has been implemented within a VR game designed to target auditory hypersensitivity in autistic people, named SoundFields [25]. During the course of game-play, players are exposed to realistic reproductions of feared auditory stimuli through the use of binaural based spatial audio. In a small scale study (n=6) conducted by the authors [25], after 4 experimental sessions participants exhibited a significant decrease in self-reported anxiety associated with feared auditory stimuli.

**Figure 1:**
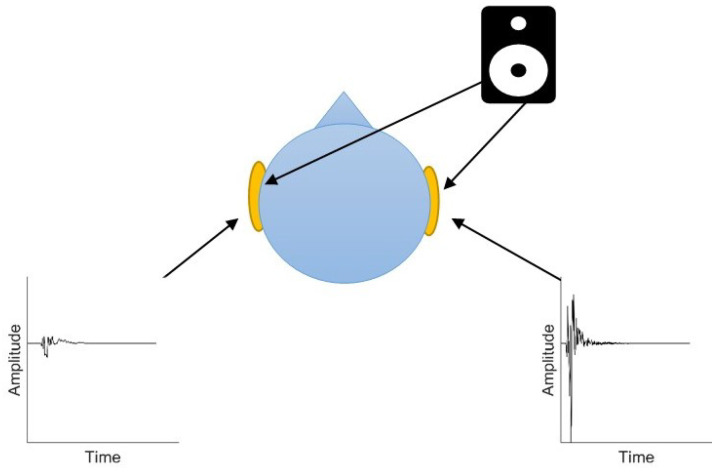
Binaural cues for horizontal localization. Plots display two time-domain representations of Head Related Transfer Functions (HRTF) recordings (45${}^{\circ }$ azimuth & 0${}^{\circ }$ elevation) extracted from the SADIE Database [27]. Reprinted with permission from [28].

Within VR applications binaural based spatial audio is utilised to render realistic auditory environments via headphones [26]. This approach to audio reproduction can simulate moving virtual sound sources which rotate and transform based upon the dynamic rotation and position of a listener’s head within a virtual space. This can be achieved through filters which simulate the free-field acoustic path of a sound source to the ear canal, know as Head Related Transfer Functions (HRTFs). HRTF’s contain the directional dependant information such as interaural differences in time and intensity and spectral shaping caused by sound waves interacting with the head, torso and pinnae (see Figure 1). Binaural rendering can subsequently be accomplished by filtering a monaural anechoic signal with the HRTF representing the desired point across a 360sphere for each ear.

Today, most VR head-mounted-displays (HMDs) make use of accelerometers and image tracking to detect force, 360orientation and measure the device’s position with a three-dimensional space. Once the audio rendering system receives the *x*, *y* and *z* directional data, it executes an interpolation between the closest HRTF pair. In addition, distance can be accurately simulated by manipulating the amplitude, frequency and reverberant energy of a sound, in response to the virtual space between the source and listener. Finally, by reproducing an auditory environment over headphones it is possible to isolate the left and right audio channels and effectively replicate the binaural cues essential for sound source localisation.

Primarily, the use of virtual reality to treat anxiety and other psychiatric disorders has relied on the accurate visual representation of the feared stimuli, with sound only playing an accompanying and lesser role [29,30]. However, when removed from accompanying visual stimuli, sound alone can also be used to induce a strong emotional response. Panksepp and Bernatzky suggest that the use of sound can have a greater neurological impact on the subcortical emotional systems more than visuals [31]. This has implications for the use of spatial audio rendering techniques which have been used to to support visual environments in virtual reality exosure therapy (VRET) interventions for combat related post-traumatic stress disorder [32], fear of moths (mottephobia) [33], fear of the dark [34] and to desensitise autistic children to airport stimuli that may cause distress [35]. However, there is limited research with mixed results investigating its capability as a primary tool for inducing the required amount of anxiety for successful exposure therapy. Brinkman et al. [36] compared the effects of different audio rendering techniques over headphones on participants’ self-reported anxiety, indicating that compared to mono, stereo and Dolby 5.1 surround, binaural 3D audio generated significantly higher levels of anxiety. Finally during the study conducted by the authors [25], participants diagnosed with ASD reported negative emotional responses whilst listening to adverse auditory stimuli rendered using binaural based spatial audio.

This paper aims to provide further evidence to support the use of binaural spatial audio as a rendering tool to aid in the reduction of auditory hypersensitivity in autistic young people. Previous work by the authors [25] indicates spatial audio can be effective for this purpose. However, here no comparison with traditional audio rendering techniques were made and the previous pilot study lacked of a control condition without intervention. In order to overcome these limitations, the current study employed a mixed crossover study design with a larger sample size. Participants were either allocated to a binaural spatial audio experimental or a stereo condition. They also participated in a control period without treatment. Subjective anxiety measures in response to target stimuli were measured before the intervention, after the crossover between experimental and control period, and at the very end of the study. Furthermore, follow up measurements were taken to evaluate generalisation.

## 2. Methods

### 2.1. Study Design

**Figure 2:**
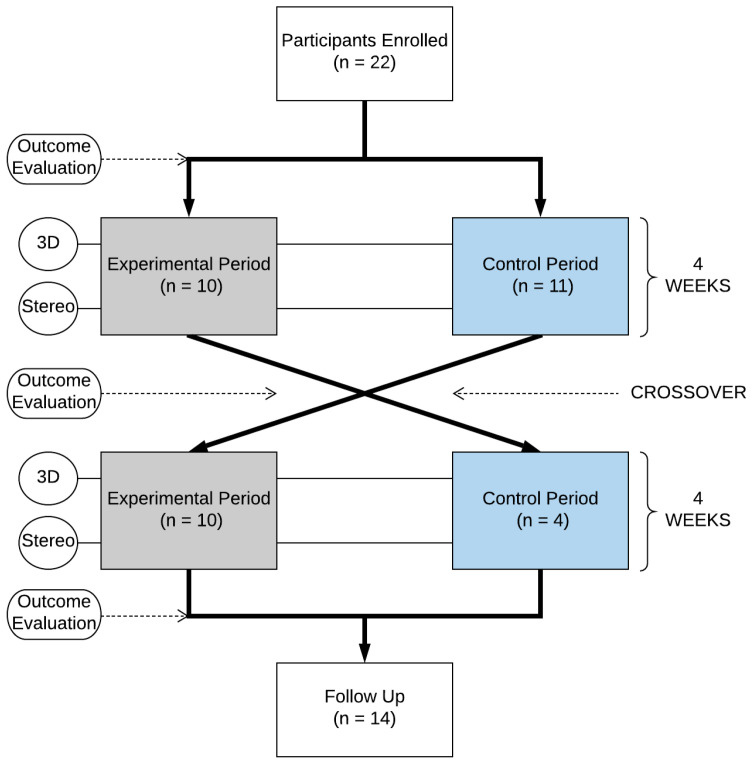
Consort diagram.

Participants were randomly allocated to one of either two experimental conditions, a 3D audio group and stereo audio group. The 3D audio group would be exposed to auditory stimuli delivered via head-tracked binuaural based spatial audio rendered over headphones. Those in the stereo audio group would listen to a head-tracked stereo audio over headphones. The investigation followed an in-between subjects crossover study design (see Figure 2). Each participant was randomly allocated to one of two study arms which would experience both the experimental and control periods consecutively [37]. The sequence in which they were exposed to these periods would be different for each arm, Arm 1: Experimental-Control, and Arm 2: Control-Experimental. During the experimental phase, each participant’s audio would be rendered using the technique based on their pre-assigned experimental group. Each period consisted of four sessions (one per week) with a duration of up to 40 min. All sessions were conducted at the school the participant was recruited at and each participant was accompanied by a member of school staff. This study and methods were approved by the University of York Department of Electronics (Johnston220219).

### 2.2. Participants

The experimental group consisted of 22 children and adolescents (18 male and 4 female, mean age = 12.23, SD = 1.56, range of 8–15 years). Participants were recruited through three special education schools located in North Yorkshire and East Yorkshire, UK. All participants had a formal diagnosis of autism spectrum disorder obtained from their local national health trust. Twenty participants displayed the social, cognitive and motor functioning abilities associated with moderate to high functioning autism, two of the participants were low functioning. Exclusion criteria were self-reported hearing problems; physical disabilities that would limit movement around experiment space; and an inability to finish the task. An experiment information pack was provided to the parent/guardian of each participant and they were required to provide information about any sounds which the participant may find either annoying or distressful.

Out of the 22 participants recruited a total 20 completed the experimental period (Group 1: n=10, Group 2: n=10), with two not being able to complete all four experimental sessions. A total of 14 participants successfully completed the control period (Group 1: n=10, Group 2: n=4). This was due to school closures put in place as a result of the COVID-19 pandemic. Participants were randomly assigned to the experimental condition based upon the order to which they arrived at the baseline measurement session.

### 2.3. Equipment

All visuals were rendered using an Oculus Rift S (https://www.oculus.com/rift-s/, Menlo Park, CA, USA) head mounted display using an MSI GP74 gaming laptop (https://www.msi.com, New Taipei City, Taiwan). Audio was delivered using Sennheiser HD 650 (https://www.sennheiser-hearing.com/en-UK/p/hd-650/, Wedemark, Germany) open back headphones. Participant head rotation and positional data within the virtual environment was tracked with 6DoF using the Oculus Rift S cameras. Participants controlled the in-game avatar using the Oculus Touch controllers.

### 2.4. Game Intervention

SoundFields is a virtual reality serious computer game aimed at children with autism experiencing auditory hypersensitivity [25]. Based on the principles of CBT, players are exposed to binaural based spatial audio reproductions of problematic stimuli via voluntary interactions with in-game mechanics. For full details regarding game-play and mechanics of SoundFields please refer to [25]. In brief, game-play is centred around finding and collecting non-playable characters (NPCs) which emit spatial audio representations during player interaction. Once successfully caught the player is rewarded with in-game currency which can be spent in a virtual shop in order to purchase aesthetic components for the player’s avatar. The game is divided into four mini-games, two of which are embedded with exposure therapy mechanics and used throughout the four week experimental period. The remaining mini-games do not render any problematic sounds and are utilised during the four week control period. Players are able to choose which game they play and can repeat it as many times as they please.

### 2.5. Experimental Procedure

#### 2.5.1. Baseline Assessment

**Figure 3:**
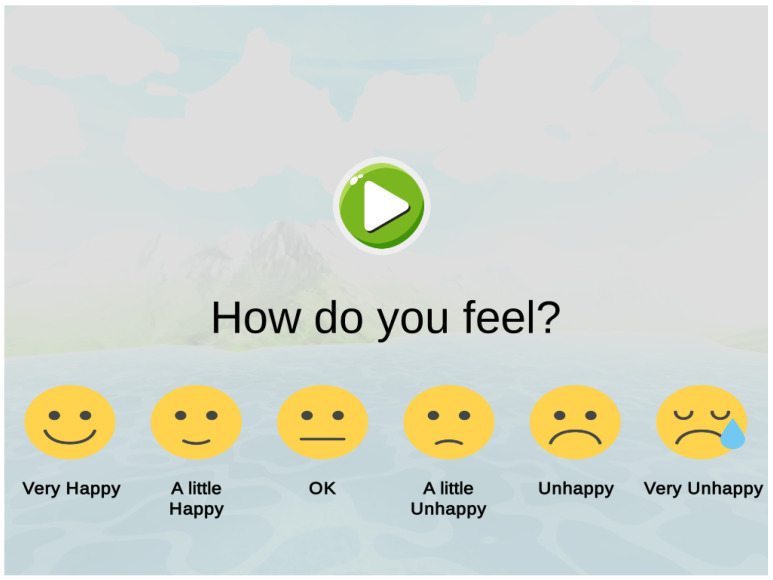
Audio Interactive Questionnaire.

Baseline measurements were recorded one week prior to the beginning of the intervention. Each participant completed an identical audio based questionnaire in which they would rate their emotional perception of specific sounds. A series of emojis were designed to translate an analogue Likert scale into graphical information that could be understood by the participant and bypass any possible communication impairments (see Figure 3). A total of twenty-two types of sounds were included, eleven representing all the problematic sounds provided through parent questionnaires, and eleven “relaxing” soundscapes taken from the Eigenscape database [38]. All audio was presented using binaural based spatial audio at the highest exposure level, simulating the smallest distance between the listener and the virtual sound source. Participant responses to the presented stimuli were recorded in Unity3D and exported as a .txt file. Finally, all participants during this session used the virtual reality equipment to become accustomed to the controls and head mounted display.

Following this session target stimuli would be allocated based upon the two stimuli with the highest self-reported emotional response values. If more than one stimuli shared the same value the stimulus would be designated using a randomly number generator.

#### 2.5.2. Session Procedure: Experimental Period

Each participant was given the opportunity to play the SoundFields game for a period of approximately 30 min, once a week over the course of four weeks. Throughout the experiment a support worker would be present to communicate with the participant and provide assistance if they became distressed. However, they were not permitted to deliver instructions. At the start of each session the investigator would select the target stimuli for the participant from a library of sounds integrated into the application. When the participant began the first session, they were told that the goal of the game is to collect orbs throughout the environment and in return they will be rewarded with one unit of in-game currency. It was also explained that golden orbs will play sounds they may find annoying or not like, but if they are successful in collecting them they will be rewarded with ten units of in-game currency. The participant would then be shown the in-game shop and explained that it is possible to purchase new aesthetic items for their in game avatar using the currency they have earned. The investigator would also tell the participant that if at any time they wanted to stop then the session would end. The session would also end if the participant became distressed or if the member of staff deemed it necessary. Throughout each session, the investigator would limit interaction with the participants to giving assistance with gaming controls.

Participants were free to move around the virtual environment and play the available mini-games as many times as they pleased. During each experimental session target stimuli were presented to the participant a maximum of 20 times through the exposure based mechanics embedded into each mini-game explained in [25]. During game-play, the software calculated the probability of each target stimulus being made accessible within the virtual environment based upon the total amount of times the participant has successfully completed an exposure mechanic task and the remaining time of the session. The total amount of time for a single interaction with these mechanics is a maximum of ≈5 s. This is based on the interaction mechanics of each experimental period mini-game.

To simulate exposure hierarchies implemented within clinical based desensitisation interventions [7], the stimulus would be played at the corresponding virtual distance (see Table 1). In addition, target stimuli would be rendered based upon their assigned experimental condition; 3D audio or stereo. From session two, virtual auditory stimuli were moved closer to the participant at the beginning of each session. However this would only be done if in the previous session the participant showed no distress, voluntarily interacted with exposure based mechanics and gave no negative feedback at the end of the session. After 4 weeks the participant would complete the audio based questionnaire.

#### 2.5.3. Session Procedure: Control Period

Sessions during the control period would follow the same format as those in the experimental period. However, the key difference would be that the available mini-games would not contain exposure based mechanics which played target stimuli. Following the four week control period participants completed the audio based questionnaire, exposed to both target and non-target stimuli.

#### 2.5.4. Session Procedure: Follow-Up Measurement

Follow-up measurement sessions took place between four and five (depending upon recruitment school schedules) weeks following the completing of the experimental period. During each session participants completed the audio based questionnaire, providing self-reported emotional responses to target and non-target stimuli.

### 2.6. Data Collection

#### 2.6.1. Primary Outcome: Self-Reported Emotional Response

A quantitative assessment was used to measure the emotional associations each participant experienced when presented with both target and non-target stimuli. Using a standalone application developed in Unity3D, participants were required to self-report their perceived emotional response towards the presented auditory stimuli. Once the survey is completed, the software exported the participant’s response to each sound in txt file format. The application utilised an analogue Likert scale between 1 and 6 depicted by simple face images and accompanied by short descriptive text, whereby 1 represented ’very happy’ and 6 represents ’very sad’ (see Figure 3). Likert scales using smiley faces have been used extensively in the subjective measurement of children’s emotional and physical responses in research [39,40,41,42,43]. By presenting a set of smiley face images this technique provides the child with an effective method to communicate their own subjective assessment of the situation or question, regardless of language or reading abilities [44]. This is therefore an effective tool for individuals living with autism who often experience difficulties in communication and emotional recognition. Importantly, this approach has been used as an assessment tool in autism research for a virtual reality intervention targeting reducing social anxiety [45] and in the investigation of SoundFields [25] and the Sinbad and the Magic Cure project [22], serious games developed to address auditory hypersensitivity in autistic children.

#### 2.6.2. Secondary Outcome: Tracked Voluntary Participant Interaction with Target Auditory Stimuli

During each experimental session data was recorded within the SoundFields application that documents voluntary interaction with target stimuli. This was achieved by measuring the total amount of time each participant engages with the exposure based mechanics of mini-game that result in the rendering of target audio stimuli. For a full description of the SoundFields mechanics and game play please refer to [25]. As mentioned in Section 2.5.2 each target stimuli was presented to participants a maximum of 20 times for approximately 5 s, therefore the highest possible value recording is ≈200 s.

Despite the extensive use of a smiley face Likert scale in research, there is a small amount of literature conducted with neuro-typical participants that observes validity issues with this technique due to issues in communication [46] and social desirability bias [47]. Taking this into account, recording voluntary interaction could bypass these challenges as well as circumventing emotional recognition problems that may occur as a result of the core symptoms associated with autism during the subject self report of emotional associations.

## 3. Results

### 3.1. Self-Reported Emotional Response

**Figure 4:**
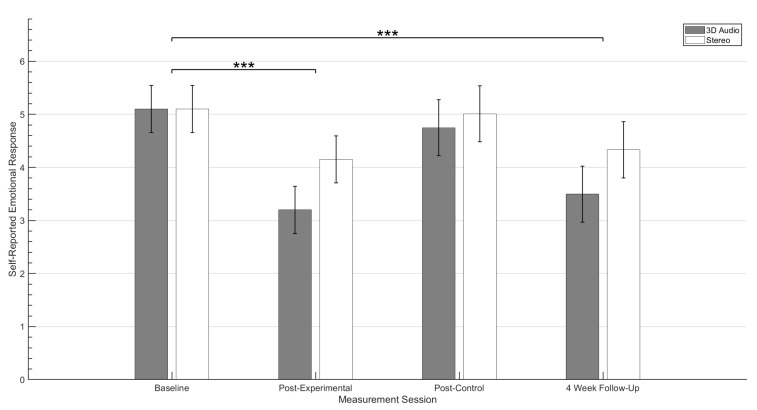
Mean self-reported emotional response to target stimuli for both experimental conditions. Whiskers denotes ±95% confidence intervals. *p* values (*** *p* value < 0.001) were determined from post hoc pairwise comparison test and are indicated above the bars.

The mean scores of self-reported emotional response (SRER) levels recorded for target stimuli across both audio conditions are shown in Figure 4. These results display a reduction in negative SRER for both experimental condition groups when comparing data collected at baseline and after the four week experimental period. A hierarchical linear model (HLM) was employed to test the effect that the experimental period, control period and audio rendering techniques had in reducing the primary outcome measurement scores for target audio stimuli. The residual covariance structure was specified as Compound Symmetry as this showed best fit to the data based on the akaike information criterion [48]. Compound symmetry structure assumes that all variances are and co-variances are consistent across the repeated measures observed throughout the study [49]. The model investigated the effects of the experimental periods and the experimental audio conditions on the self-reported anxiety in response to target stimuli. This was based on SRER values recorded at each outcome measurement session.

Statistical analysis indicates that experimental periods had a significant effect on the overall reduction in SRA values across both experimental conditions (F(3,45.907)=20.547, p<0.001). Further post hoc pairwise comparison between baseline and post-experimental period scores also revealed a significant reduction in estimated marginal means (p<0.001). In addition, no significant differences were observed between self-reported anxiety levels recorded at baseline and post-control period sessions (p=1.000). Finally, no significant changes were observed in SRER values recorded between the post-experimental period and the follow-up measurement session. This suggests that any reduction in anxiety associated with target stimuli across both conditions was maintained for at least 4 weeks.

In regards to the effect of the experimental condition, HLM analysis indicates that the use of binaural based spatial audio does have a significant effect upon reducing the participants self reported anxiety towards the target auditory stimuli presented within the virtual reality environment (F(1,17.773)=6.783, p=0.018).

**Figure 5:**
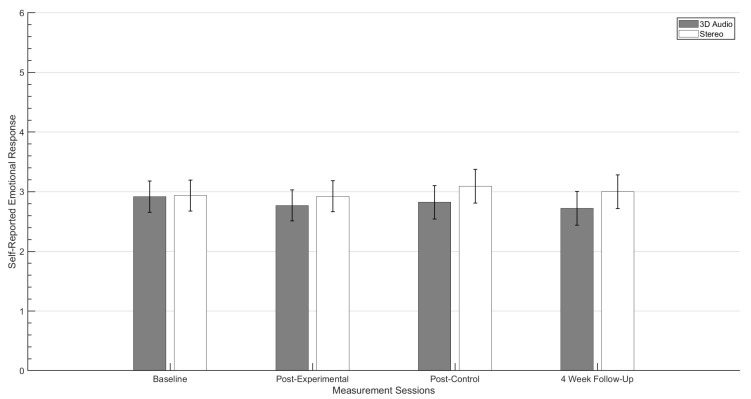
Mean self-reported emotional response to non-target stimuli for both experimental conditions. Whiskers denotes ±95% confidence intervals.

Figure 5 shows SRER levels for non-target stimuli recorded for both experimental conditions across the entire investigation. The plot shows for both groups very little difference between the levels values across all four measurement sessions. In addition, non-target stimuli data was analysed using the same statistical tests as the target stimuli. For both audio conditions there was no significant effect of the experimental periods on the SRA values (F(3,41.670)=0.838, p=0.481). Furthermore, HLM analysis revealed that the audio rendering condition had no significant effect on the SRA values for non-target stimuli over the experimental period (F(1,17.027)=1.269, p=0.276).

Descriptive statistics were used to examine the mean changes in SRA scores for each individual target stimuli between the pre- and post-experimental period measurements. It is important to note that although the sirens stimuli was used as a target stimuli it was done so for only one participant in the stereo condition, therefore it has been omitted from this analysis.

In reference to Table 2, it can be seen that across all stimuli, with the exception of ‘fireworks’, the 3D audio group experienced the greater increase in changes between self-reported anxiety values between the baseline and post experimental assessments. Furthermore, Table 2 shows the mean baseline, and post-experimental self reported anxiety figures for each target stimulus alongside the corresponding percentage difference in SRER scores across each audio condition. It can be seen that the experimental period had a greater positive impact on those participants exposed to the ‘children playing’ stimulus in the 3D audio group with a decrease of SRA of 50%, this was followed by the ‘children fighting’ stimulus which showed a 33.18% decrease. In comparison, participants in the stereo condition experienced some of the lowest decreases in anxiety, ‘children playing’ experiencing a decrease of 3.77% and ‘children fighting’ anxiety levels reducing by 9.52%.

### 3.2. Tracked Interaction Times

A HLM was used to investigate the effect of the experimental period and audio rendering techniques on the total amount of time participants voluntarily interacted within-game mechanics that delivered target auditory stimuli. This was compared between both experimental groups and across the four week experimental period. The covariance structure for this model was specified as Compound Symmetry.

**Figure 6:**
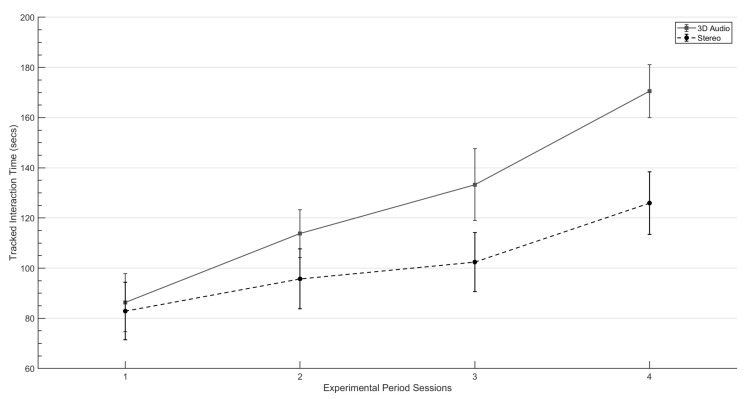
Mean tracked interactions times across all experimental sessions. The whiskers denote standard-error.

In Figure 6, it can be seen that the amount of tracked interaction time for both the 3D audio and stereo groups increases across the four week experimental period. HLM analyses show that the subsequent experimental sessions had a significant effect upon tracked interaction times (F(58)=46.361, p=0.001) for both audio conditions. In addition, this plot shows that participants in the 3D audio group did voluntarily interact more with target audio stimuli than those in the Stereo group. Statistical analysis showed there was a significant interaction between the audio rendering technique and the experimental period (F(58)=3.825, p=0.05).

Further analysis was conducted on each individual experimental session, comparing tracked interaction times between experimental conditions. An independent samples *t*-test that indicated no significant difference between the 3D audio (M=90.33, SD=36.39) and stereo groups (M=84.89, SD=40.13); (t(18)=0.317, p<0.755) during Session 1. Similar results were also identified for Session 2 in which no significant effect was recorded (t(18)=1.523, p<0.145) despite the increased mean difference between the 3D audio (M=116.66, SD=32.59) and stereo (M=92.33, SD=12.20) groups. In Session 3 however, the difference between the two groups is significant (t(18)=2.218, p<0.040). Additionally, in Session Four the 3D audio group (M=163.05, SD=35.51) can be seen interacting more with target stimuli than the stereo condition (M=126.95, SD=39.88) (t(18)=2.138, p<0.047).

## 4. Discussion

The main objective of this study was to compare binaural based spatial audio to stereo rendering as an approach to delivering audio stimuli in an exposure training VR game targeting auditory hypersensitivity in autistic participants. In line with the outcomes of previous research [22,25,50], results from this investigation support the use of exposure based training within a serious game environment in reducing self reported anxiety associated with specific environmental sounds for autistic individuals. What further reinforces these findings is the implementation of the four week control period into the experimental design. Across both audio conditions there were no significant changes in SRER values between measurements collected before and after the completion of the control sessions. Moreover, no significant changes in values were reproduced for non-target stimuli. How the participants felt about non-target sounds remained reasonably consistent across the entirety of the eight week program. Taking into account the results from both the control period and non-target sounds, it could be considered that any impact on self-reported emotional response is specific to the exposure based game-mechanics and not a general training effect.

The comparison of audio rendering techniques was the primary objective of this paper. According to the statistical analysis of SRER scores, those participants listening to averse stimuli delivered via binaural based spatial audio showed a significantly increased improvement in self-reported anxiety than those in the stereo rendering condition following the four weekly experimental sessions. Furthermore, the appreciable improvements of the participants in the 3D audio group cannot be attributed to differences in baseline measurements. Statistical analysis showed that the baseline anxiety scores for these two groups were not significantly different.

When examining these results it is important to consider again the use of VR therapy to reduce specific phobias in individuals with and without autism spectrum disorder [24,51]. Firstly, this technology has the ability to accurately and safely simulate a controlled three-dimensional environment to create a bespoke exposure therapy experience according to the user’s needs [52]. However, what separates VR from flat-screen solutions is the sense of presence felt by the user, and it is presence that is considered an important component in activating the relevant fear structures to achieve habituation [53]. Furthermore, it has already been observed that autistic children experience similar levels of presence as their typically developed peers within virtual environments [54]. Secondly, but more crucial to the application of this research, is the role of spatial audio in influencing the sense of presence in VR. Realistic auditory environments rendered through spatial audio have been shown to increase levels of presence [55,56,57,58]. Furthermore, similar spatial audio rendering techniques to those used in this study have been observed to elicit feelings of fear and anxiety [59,60]. Consequently the results of this study could be expected. Those diagnosed with autism often experience difficulties with imagination [61,62] which leads to a need for stimuli to be contextualised. Therefore, participants in the 3D audio condition who experience a sound closer to what they hear in the real world in terms of localisation, dynamic movement and environmental acoustic characteristics should feel increased similarity between the real and virtual stimulus over those in the stereo audio group. This familiar interpretation of virtual stimuli rendered within virtual environments by young people with ASD has been also attributed to the successful outcomes of past VR based interventions [63,64,65]. Further to this, White et al. [17] notes that autistic people require frequent practice with contextualised exposure in order to increase the chances of reducing anxiety associated symptoms. This could serve as a justification for the larger decreases in SRER displayed by participants listening to the ‘*Children Fighting*’ and ‘*Children Playing*’ target stimuli compared to those listening to ‘*Fireworks*’ or ‘*Alarms*’ in the 3D audio group. This study was carried out within the school each participant was recruited during active school days. Consequently in addition to the repeated exposure to these stimuli with the virtual reality game, the opportunity for real-world exposure of these environmental soundscapes is much higher within the school environment than the other target stimuli such as the ‘*Fireworks*’.

By recording the voluntary exposure to target stimuli during each experimental session it is possible to measure any increase in tolerance towards a participants target sounds to support measured values of self-reported emotion response. When examining the results collected across the completed experimental period, Figure 6 demonstrates that the 3D audio group spent longer times interacting with stimuli than those in the stereo group. However, these changes were not statistically different. This is most likely explained by the similar times recorded by both groups during the first experimental period session. The lack of significant differences recorded in Session 1 in terms of the mean and distribution of values could be interpreted in two ways. First, the participants would be experiencing a new virtual environment with new mechanics and so lower times could correspond to periods exploring and experimenting with the VR environment and game. Subsequently spending less time interacting with exposure based mechanics. Secondly, this would be the first time each participant heard their target sounds outside of the outcome measurement sessions, therefore are less likely to want to engage. However, comparing tracked interaction times between the two groups on a session by session basis does reveal some statistically significant differences from the third session, with the 3D audio group interacting more with the game mechanics that deliver problematic sounds. It could therefore be accepted that these results mirror the larger decreases in self-reported anxiety values between the baseline and post-experimental session measurements. Finally, one crucial feature of these results is that each session represents an increase in the exposure hierarchy with the virtual sound source being moved closer to the participant, resulting in an increase in perceptual loudness. Therefore, an increase in voluntary interaction with a sound that the participant has personally reported a negative emotional association with makes these results more meaningful. As increases in tracked interaction times and decreases in self-reported anxiety values were recorded across both audio conditions, it is important to consider the impact game-play and the virtual environment had on the outcomes of this study.

In parallel to the advantages of using 3D audio as a technique to simulating realistic sound environments that have been identified in this study, the context in which spatial sound is delivered is also a key advantage of the format. Computer games have been successfully used in the past to target auditory hypersensitivity in autistic young people [22,50]. However as stated in [25], SoundFields is the first game in this context that uses virtual reality. The levels of engagement with the SoundFields game displayed by participants is consistent with those in the previous work [25], however results from the current study could be considered more meaningful due to the larger sample size of this investigation. Across both audio conditions all participants with the exception of two completed the four weekly experimental sessions, with six not finishing the control period due to school closures. Alongside the quantifiable results measuring engagement with exposure based mechanics described above, statements were also provided by staff from the recruitment schools. It was noted that participants were ‘highly motivated’ and ‘excited’ to begin their weekly virtual reality sessions. Further to this, staff observed that participants maintained interest during the 8 week study despite some being expected to become disinterested as the study progressed. These comments and the continued interaction with exposure mechanics recorded in-game during this study, are corroborated by literature acknowledging that computer game based interventions are enjoyed by autistic individuals [21,22,50,66,67]. The study provides evidence that exposure based therapy embedded into VR based computer game mechanics can improve self-reported negative emotions associated with problematic sounds. The voluntary interaction with target stimuli supported by the intrinsic reward system aimed to develop the participant’s confidence and create positive associations with the stimuli. Both primary and secondary outcomes of this investigation indicate that this applied to participants across both experimental groups.

Despite the positive results from this study there are a number of limitations that must be considered. Firstly, 30% (n=6) of the participants were unable to complete the the control period due to the school closures implemented during the COVID-19 pandemic. This reduces the statistical power of the results that validate the experimental sessions as the primary influencing factor of the investigation. Secondly, the study would have benefited from information collected from parents and staff regarding how participants reacted to real-world target stimuli outside of the virtual reality environment. This could be evaluated comparing pre- and post-intervention measurements using the Short Sensory Profile [68] and parental questionnaires such as the Parent Stress Index [69].

## 5. Conclusions

This paper presented an experiment designed to evaluate the use of binaural based spatial audio to address auditory hypersensitivity experienced by autistic young people compared against a stereo audio rendering approach. Self-reported levels of emotional response were used as the primary outcomes to quantify any changes in tolerance towards target and non-target audio stimuli following the four week experimental period. In addition, data was also gathered during each experimental period session which recorded the total amount of time each participant voluntarily interacted with in-game mechanics that delivered target auditory stimuli.

Results coincide with previous research that indicates spatial audio rendered used within a VR exposure game can successfully aid in decreasing negative emotions associated with averse environmental auditory stimuli [25]. This suggests a reduction in auditory hypersensitivity based reactions presented by the autistic participants. Furthermore, those listening to spatial audio simulations showed significantly more improvement than those listening to stereo renders. However, the use of spatial audio can extend further than the realistic reproduction of problematic sounds. Today, VR and consumer mobile technology is capable of rendering spatially accurate virtual auditory environments that can respond to 6 Degrees of Freedom. This presents the opportunity to increase accessibility to therapy, allowing interventions to be conducted within home or school environments. This not only improves motivation to engage with therapy, but also places the individual within the environment they are most likely to experience averse sounds, resulting in further contextualisation of the presented stimulus and the potential strengthening and generalisation of therapeutic outcomes.

## Figures and Tables

**Table 1:** Exposure hierarchy. Each exposure level corresponds to the distance between the participant and the virtual sound source; distance is represented in metres.

Exposure Level	Virtual Distance between Player and Stimulus
1	25 m
2	15 m
3	5 m
4	2.5 m

**Table 2:** Changes in mean self-reported anxiety scores for target stimuli across both experimental conditions, showing pre and post measurement scores with percentage decrease.

Condition	Target Stimulus	Participants (n)	Pre-Test (*M*)	Post-Test (*M*)	% Decrease
	Alarm	2	4.75	3.25	31.59
	Baby	2	5	4.25	10.53
	Engine	2	5	3.25	35
3D Audio	Fireworks	2	5.5	4.5	18.18
	Hair Dryer	1	6	4	33.33
	Children Fighting	4	5.12	3.37	34.18
	Children Playing	7	5	2.5	50
	Alarm	4	5.25	4.37	16.76
	Baby	2	5.5	5	9.09
	Engine	2	5.25	4	23.81
Stereo	Fireworks	3	5.33	3.67	31.14
	Hair Dryer	3	5	5	20
	Children Fighting	2	5.25	4.75	9.52
	Children Playing	3	4.5	4.33	3.77

## Data Availability

The data that support the findings of this study are available from the authors upon reasonable request.

## Abbreviations

The following abbreviations are used in this manuscript:

ASD	Autism Spectrum Disorders
HMD	Head Mounted Display
NPC	Non-Playable Character
SG	Serious Game
VE	Virtual Environment
VR	Virtual Reality
